# Evaluating evidence-based recruitment strategies for Alzheimer’s disease and related dementias clinical trial research: A literature review

**DOI:** 10.1016/j.tjpad.2026.100532

**Published:** 2026-03-14

**Authors:** Mireille Jacobson, Christina Deuschle, Desi Peneva, Alice Nuo-Yi Wang, Cooper Roache, Meghan Walsh, Phyllis Barkman Ferrell, Maria-Alice Manetas, Rema Raman, Paul Aisen, Dana Goldman

**Affiliations:** a Leonard D. Schaeffer Center for Health Policy and Economics, University of Southern California, Los Angeles, CA, USA; b Leonard Davis School of Gerontology, University of Southern California, Los Angeles, CA, USA; c Rose Li and Associates, Bethesda, MD, USA; d Price School of Public Policy, University of Southern California, Los Angeles, CA, USA; e Alzheimer’s Therapeutic Research Institute, Keck School of Medicine, University of Southern California, San Diego, CA, USA

**Keywords:** AD/ADRD clinical research, Recruitment, Research participation, Reporting transparency

## Abstract

**Background:**

With the prevalence of Alzheimer’s disease and related dementias (ADRD) rising, prevention and treatment clinical trials are increasingly important. Yet inadequate participant recruitment to ADRD research—a leading cause of trial delays, suspensions, or discontinuations—continues to hinder innovation and increase costs. This literature review aimed to identify ADRD recruitment strategies that are effective and ineffective, based on quantitative outcomes, with the goal of optimizing recruitment and advancing recruitment science.

**Methods:**

PubMed, Google Scholar, relevant ADRD websites, and references were searched for studies meeting inclusion criteria—those reporting quantitative outcomes aimed at improving recruitment rates, timely recruitment, or representation. Reference lists from relevant systematic reviews and meta-analyses, including publications from leading researchers, were also examined. The Mixed Methods Appraisal Tool (MMAT) was used to assess evidence quality.

**Results:**

The search yielded 965 publications, of which 50 met inclusion criteria. Few studies reported recruitment methods for pharmacological trials, and many lacked sufficient detail or standardized reporting to assess quality using MMAT criteria, making it difficult to determine effectiveness. Recruitment efforts deemed successful by study authors often relied on multi-pronged approaches integrating community engagement, structured outreach, and digital tools. However, evidence for effective and scalable strategies remains limited.

**Conclusion:**

Advancing ADRD recruitment science requires progress in two key areas: embedding recruitment evaluation directly into trial protocols and encouraging broader sharing of recruitment data. Routine practices, such as publishing recruitment outcomes and adopting standardized reporting, can help close the evidence gap. These approaches enable comparison, replication, and generalizability of effective strategies, ultimately accelerating progress in ADRD research.

## Highlights

• Identifies recruitment challenges limiting efficient and representative ADRD trials.
• Summarizes quantitative evidence on effective and ineffective ADRD recruitment strategies.
• Finds that multipronged recruitment approaches show promise.
• Reveals limited evidence and lack of standardization for scalable recruitment.
• Emphasizes routine, transparent reporting to advance science of ADRD recruitment.

## 1 Introduction

With increasing life expectancy, the prevalence of Alzheimer’s disease and related dementias (ADRD) in the United States is projected to continue rising [[1], [2], [3]]. Although recently approved disease-modifying therapies provide benefits to individuals with sufficient amyloid burden and access to necessary clinical infrastructure [4,5], the need for novel ADRD treatments remains substantial. Meeting this need, however, is hindered by persistent recruitment shortfalls: of the 164 ADRD-related clinical trials underway in 2024, most report challenges in participant enrollment [6,7]. Insufficient enrollment is cited as one of the leading causes of trial suspension, termination, or discontinuation across study types [8], increasing research costs and slowing innovation [9]. Identifying effective recruitment strategies to ensure timely enrollment is critical for developing novel therapies that benefit all people living with ADRD [10,11].

Recruitment into ADRD research is impeded by numerous barriers, including restrictive eligibility criteria, the need for invasive procedures to confirm diagnosis and study eligibility, the requirement to enroll a study partner (e.g., a caregiver or family member), and significant demands on participant’s time, such as frequent travel to study sites, limited awareness of early Alzheimer’s disease (AD) symptoms, and fear of receiving an AD diagnosis [[11], [12], [13], [14]]. These challenges contribute to clinical trials and other studies that do not accurately reflect the ADRD population. Racial and ethnic groups, including Black/African American, Hispanic/Latino, and Asian Americans populations, are underrepresented in ADRD research [11,14,15], despite facing a higher risk of dementia than White Americans [6,15]. Other high-risk groups, such as rural residents, socioeconomically disadvantaged individuals, and veterans, are similarly underrepresented [16,17]. Further, clinical studies tend to overrepresent individuals with higher levels of education, while individuals from rural or socioeconomically disadvantaged communities tend to be unrepresented [11]. This lack of representativeness limits the generalizability and applicability of clinical trial findings.

This review synthesizes the existing literature on evidence-based recruitment strategies in ADRD clinical research. Given the substantial heterogeneity in study design, recruitment approaches, and outcome reporting across studies, the literature does not lend itself to meta-analysis. Instead, we focus on identifying and characterizing studies that report quantifiable outcomes, with particular attention to the quality of evidence. This approach allows us to highlight strategies with promise for improving trial recruitment and to identify avenues for advancing the science of recruitment in ADRD research.

ADRD clinical trials and research studies use a range of recruiting strategies, often in combination, including healthcare provider (HCP) referrals, community engagement (CE) and outreach, registry and matching services, paper mailings and flyers, phone calls, door-to-door recruiting, and media campaigns (e.g., radio, newspaper) [14,[18], [19], [20], [21]]. Despite these efforts, recruitment barriers persist. For example, HCP referrals, considered a reliable source for participant enrollment, is constrained by overburdened clinician workflows, insufficient clinical tools for ADRD diagnosis, provider concerns that patient risks may outweigh trial benefits, and lack of awareness about ongoing clinical trials [14,22].

In response to these challenges, the National Institute on Aging (NIA) released *the National Strategy for Recruitment and Participation in ADRD Clinical Research* in 2018, emphasizing the need to build an “applied science of recruitment” through evidence-based approaches to evaluate and improve participant enrollment and retention in both clinical and observational research settings [15]. Since then, ADRD research has made progress in recruiting representative participants through initiatives such as the Recruitment, Engagement, and Retention Unit of the Alzheimer’s Clinical Trials Consortium (ACTC, an NIA-funded consortium conducting ADRD trials), which uses a data-driven, evidence-based, participant- and site-focused approach to recruitment and retention; centralized participant registries such as the Trial-Ready Cohort for Preclinical/Prodromal Alzheimer’s Disease (TRC-PAD) [23] and the Alzheimer’s Prevention Registry [24]; prescreening participant data collection; and innovative decentralized recruitment practices such as plasma prescreening and remote screening [[25], [26], [27], [28], [29], [30], [31]].

Central to recruitment science is the systematic study, documentation, and evaluation of recruitment and retention methods [32,33]. Several reviews of recruitment practices have noted inconsistencies in outcome reporting, which hampers cross-study comparisons of recruitment strategies and limits the quality and quantity of evidence [18,34,35]. Equally important is reporting of both successful *and* unsuccessful recruitment efforts, which enables the replication of effective strategies and the refinement of those that fall short. Consequently, researchers have called for more robust evidence on ADRD recruitment strategies and standardized reporting of recruitment outcome measures [34,35]. This review addresses these gaps by synthesizing studies that report quantitative recruitment outcomes and assessing their quality, thereby strengthening the evidence base for ADRD recruitment science through documentation of current reporting practices, contextualization of progress, and identification of areas in need of improvement.

## 2 Methods

### 2.1 Study selection

**Fig. 1:**
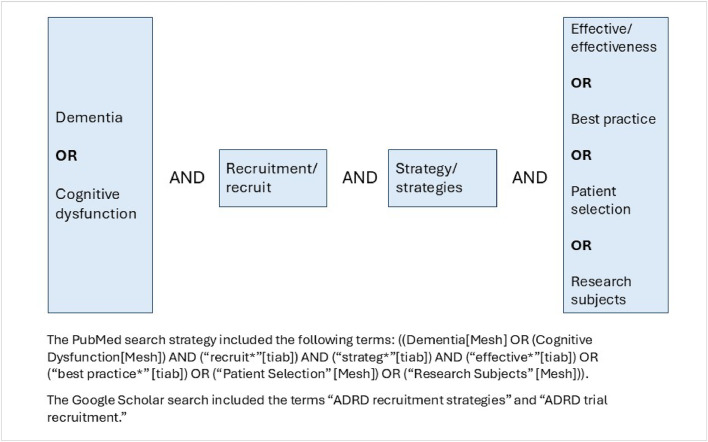
Search terms.

The PRISMA Extension for Scoping Reviews (PRISMA-ScR) guided the search process for this review [36]. The review included published peer-reviewed and grey literature examining effective and ineffective recruitment strategies for ADRD clinical research. Only studies reporting quantitative outcomes were included. Studies of interest included those aimed at improving recruitment rates, enhancing timely recruitment, and increasing participant representation and inclusion. The review was limited to English language studies conducted in U.S. populations and published between January 1, 2010, and June 30, 2025. PubMed, Google Scholar, and a convenience sample of 26 relevant ADRD websites were searched, including official websites of government agencies, research institutions, nonprofit organizations, private industry, and contract research organizations (see Fig. 1 for search terms and **Supplementary Table 1** for searched ADRD websites). A search of reference lists from relevant systematic reviews and meta-analyses, as well as citation searches of included papers and publications by leading ADRD authors based on citation rates, was also performed.

### 2.2 Data extraction

Search results were imported into Covidence, a web-based software platform used to manage systematic reviews. To ensure accuracy of the review, two reviewers screened titles and abstracts and then proceeded to a full-text review of the remaining articles, with conflicts resolved by a third reviewer. A standardized data extraction form was created for both the published and grey literature and completed by two reviewers, with periodic consultation from a third reviewer as needed. Extracted information included measures such as total number or percentage of participants recruited, recruitment cost, yield rate, conversion rate, and recruitment rate. Yield rate was defined as the percentage of individuals contacted to participate who were screened; conversion rate as the percentage of individuals screened who enrolled; and recruitment rate as the number of participants recruited on average per month at a single site. When studies provided raw data without calculated rates, these rates were calculated. Additional data extracted included study design, descriptions of study participants and inclusion criteria, recruitment approach, study aims, study setting, and key findings.

### 2.3 Study quality appraisal

Evidence quality was assessed using the Mixed Methods Appraisal Tool (MMAT), Version 2018 [37]. The MMAT, commonly used in systematic reviews, is a validated instrument designed for evaluating study quality across five types: qualitative research, quantitative randomized controlled trials, quantitative non-randomized studies, quantitative descriptive studies, and mixed methods studies [38]. Studies were first evaluated using two screening questions: [1] Are there clear research questions? [2] Do the collected data allow us to address the research questions? Studies meeting screening criteria were then assessed (47 of 50). Three published poster presentations did not meet screening criteria but were retained in the synthesis because they satisfied the review’s inclusion criteria. Studies were then assessed according to type (e.g., qualitative, quantitative randomized, etc.) using a corresponding set of five quality criteria from the MMAT Version 2018 User Guide [37]. Each of the five MMAT criterion for a given study type was rated as either “yes,” “no,” or “cannot tell,” with “cannot tell” indicating insufficient information provided to determine a result. MMAT scoring guidelines enable the calculation of an overall score, the percentage of criteria met, and an aggregated score presented on a 1-5 scale, where each point represents one criterion met (1 = 20% criteria met, 5 = 100% criteria met). For each criterion, only a “yes” rating adds one point to the overall score [37,38]. For example, a study receiving “yes” rating for three criteria, “no” for one, and “cannot tell” for one, would have an overall quality score of 3 out of 5, equivalent to 60% of criteria met.

## 3 Results

### 3.1 Study characteristics

**Fig. 2:**
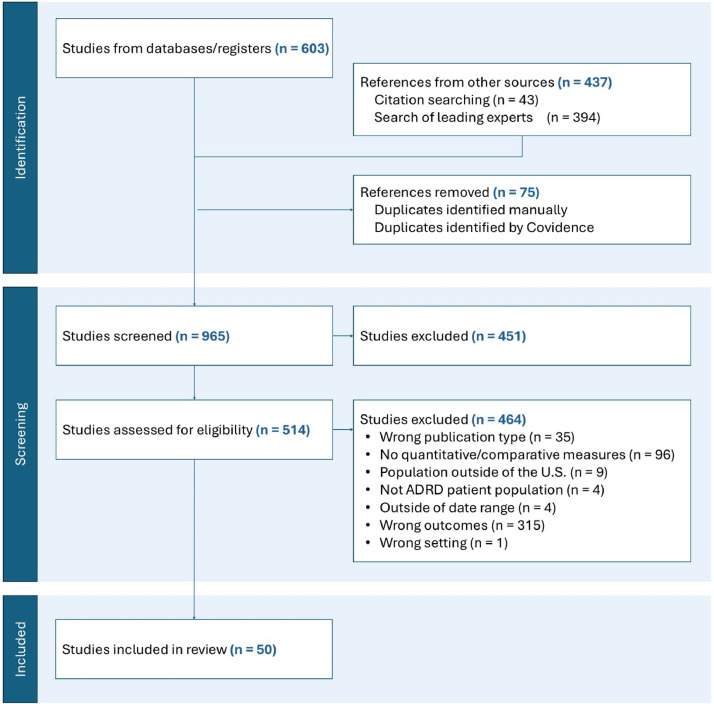
Study screening and selection process.

The initial PubMed and Google Scholar search yielded a total of 603 articles; the snowball review of identified literature reviews, citation searching, and a search of articles by leading experts in the field yielded 437 articles. 75 duplicate references were removed, and 965 studies were screened. The review of pre-identified organizations did not yield any eligible articles. 451 studies were removed during title and abstract screening, and 541 studies were assessed for eligibility. 464 studies were excluded, resulting in 50 studies included in the review (Fig. 2).

All included studies examined recruitment as a primary focus, except one [39], which analyzed claims data linked to a cohort to study ADRD incidence and discussed recruitment to that cohort. The 50 included studies were classified into three categories based on recruitment goals: broad or general recruitment efforts (n=20), recruitment of underrepresented groups (n=20), and recruitment into registries (n=10). For ease of exposition, these three categories are used to organize and synthesize the review findings.

None of the included studies reported prospective analyses from randomized controlled trials designed to evaluate ADRD recruitment strategies; rather, all recruitment studies captured in the search were observational. Many studies presented ad hoc descriptive analyses of recruitment efforts (n=19), mostly in non-pharmacological clinical trials (n=15) [21,[40], [41], [42], [43], [44], [45], [46], [47], [48], [49], [50], [51], [52], [53]]. Only four studies reported recruitment strategies from pharmacological trials [26,[54], [55], [56]]. The remaining studies reported recruitment strategies for non-clinical trial observational studies (n=10) [39], [53], [54], [55], [56], [57], [58], [59], [60], [61]; for registries (n=11) [29,[62], [63], [64], [65], [66], [67], [68], [69], [70], [71]]; did not provide enough information to determine whether a clinical trial was conducted or its type (n=8) [31,[72], [73], [74], [75], [76], [77], [78]]; or reported on intervention studies not registered as clinical trials (n=2) [79,80].

Most studies employed multi-pronged recruitment strategies (n=32), often involving outreach via diverse channels, strategic partnerships, and community engagement (CE); however, comprehensive descriptions of these strategies were inconsistently reported. CE encompasses a broad range of proactive engagement activities in community settings—such as health fairs, senior centers, faith-based organizations, or community group meetings—whereby researchers provide information about ADRD research to potentially eligible individuals. Unlike other strategies, CE emphasizes face-to-face interaction within trusted community spaces rather than indirect communication.

Although most studies were conducted by academic investigators, details on study location were limited, with little information on whether recruitment occurred in industry or academic settings. Participant enrollment numbers were reported in 94% of studies, making it the most frequently documented metric. Other key indicators, such as recruitment rate, conversion rate, and yield rate, were less commonly reported, appearing in 4%, 14%, and 18% of studies, respectively (see **Supplementary Table 2**).

Most studies (65%) did not report the time, labor, or material costs of recruitment. Of those that reported costs, two studies provided total recruitment costs, omitting the breakdown of cost per recruitment strategy. Further, studies varied in their reporting approach - some presented total costs per strategy while others reported cost per participant enrolled. Detailed and standardized information on recruitment costs and time is needed to more fully compare approaches.

### 3.2 Synthesis of results

#### 3.2.1 General recruitment strategies

Twenty of the 50 studies (40%) reported recruitment methods and outcomes for person(s) living with dementia (PLWD) and PLWD-caregiver dyads without focusing on specific demographic or underrepresented subgroups (Table 1). Most of these studies (n=13) employed multi-pronged recruitment strategies, with several studies reporting the effectiveness of individual approaches, such as the use of registries, social media, and CE to recruit participants. More than half of these articles (n=12) reported recruitment outcomes across multiple ADRD studies [26], [31], [39], [40], [46], [47], [54], [71], [72], [78], [81], [82], while the remainder (n=8) discussed recruitment outcomes for specific ADRD clinical studies [42], [43], [45], [50], [52], [53], [80], [83]. Multi-pronged recruitment approaches commonly included some combination of CE (n=13), paper mail (n=8), other print media (e.g., flyers, brochures, newspapers, magazines; n=5), HCP/word of mouth (WoM) referrals (n=8), social media campaigns (n=6), and registries (n=5).

**Table 1:** Study characteristics.

Category 1: Studies evaluating effectiveness of recruitment to ADRD research generally				
Author, Year	Objective	Recruitment Strategies	Outcomes (# or % participants enrolled by strategy)	Results
Austrom et al., 2010	Increase research participation of 4 ADRD studies	phone (helpline), print information	phone (helpline): 2 enrolled, print information: 0 enrolled	Helplines can be used as a strategy to recruit participants
Birnbaum et al., 2024	Investigate effectiveness of study referrals from the C2C registry	registry	2,805 unique individuals enrolled in studies (28.9% of referrals)	The C2C registry was a successful recruitment strategy
Birnbaum et al., 2025	Investigate effectiveness of study referrals from the C2C	registry	3,843 unique individuals were referred to 57 studies 26% of referrals led to enrollment into studies (1,642)	The C2C registry was a successful strategy
Carr et al., 2010	Compare recruitment via CE vs. HCP referrals	CE, HCP	CE (health fair): 100% HCP (nurse practitioner): 0% cost per participant: $55.18	CE was substantially the most effective strategy
Cowie & Gurney, 2018	Recruit participants into a Phase 1 clinical trial	social media	social media: 86.7% increased recruitment overall click-through rate: 3.37%, cost: $1.23 per engagement	Social media advertising was a successful and cost-effective strategy
Greimel et al., 2022	Recruit participants to FIT-AD Trial	CE, WoM, HCP, paper mail, social media, registry, press releases, flyer/brochure, newspaper/magazine, website	CE: 35.6%, $734/participant HCP and WoM: 28.1%, flyers/brochures: 12.5%, $625/participant paper mail: 8.3%, $290/participant ads: 3.1%, $1719/participant study website, social media, registry: 3.1%, $225/participant media press: 1%, $270/participant recruitment rate: 2.15 yield rate: 76.2% conversion rate: 31.9%	CE was the most effective, and second most costly strategy
Keohane et al., 2023	Examine ADRD incidence via Medicare claims	paper mail, community health centers	CHCs: 85%, paper mail: 15%	Recruiting from CHCs was substantially the most effective strategy
Kirn et al., 2023	Evaluate recruitment strategies using a centralized prescreening database (DART)	social media, registry, national campaign, local campaign, websites, referrals	websites: 50.91%, local campaign: 28.85%, referral: 8.54%, registry: 7.47%, social media: 2.8%, national campaign: 1.75%, unknown: 2.8%	Across 7 study sites, websites were the most effective strategy
Morrison et al., 2016	Recruit dyads to a randomized trial	CE, paper mail, newspaper ads	paper mail: 57%, $63/dyad newspaper: 26%, $224/dyad CE: 17%, $350/dyad overall cost/dyad: $154	Paper mail was the most effective and least costly strategy
Melikyan et al., 2019	Examine effectiveness of recruitment strategies to the 90+ study	CE, earned media, paper mail, referrals (collaborating investigators)	CE: 33.8%, $14,786 total ($261/participant) earned media: 21.4%, $4,093 total ($132/participant) paper mail: 16.6%, $6191 total ($258/participant) referrals: 15.8%, unknown: 12.4%	CE was the most effective but most costly strategy
Naylor, 2024	Examine effectiveness of recruitment strategies in ACHIEVE trial	CE; WoM; paper mail, print media, broadcast media, web-based media	broadcast media: 56.2%, print media: 41.7%, WoM: 34.2%,other strategies (NR)	Broadcast media was the most effective strategy
Peavy et al., 2020	Recruit older adults into ADRD research	CE (memory screening)	CE: 19% of those screened	CE via memory screening clinics was an effective strategy
Shadyab et al., 2021	Recruit adults with amnestic MCI into EXERT trial	CE, paper mail, registry, memory clinic rosters, EHRs, news broadcasts, PSAs, local ads	paper mail: 52%, memory clinic rosters, EHRs, registry (combined total): 25%, WoM: 6.1%, CE: 5.4%, paid advertising: 5.4%, news media: 5.4%, all other: <2% total cost per participant: $1,439 conversion rate: 29.8%	Targeted paper mailing was the most effective strategy
Szabo-Reed et al., 2023	Examine effectiveness of recruitment methods for clinical trial	CE, registry, social media, emails, website, marketing interviews/articles/editorials, print advertising	emails: 20.9%, interviews/articles/editorials: 18.7%, print advertising: 18.7%, unknown: 17.9% conversion rate: 63.73%	Across 4 study sites, emails were the most effective strategy
Teano et al., 2024	Examine recruitment outcomes from a social media campaign	social media	referred to ongoing research study: 61%	Social media was an effective strategy for referred participants to research studies
Walter et al., 2020	Recruit participants to the APT Webstudy	CE, registry, social media, earned media	registries: 69.69%, other strategies (NR)	The use of registries was an effective strategy
Walter et al., 2024	Recruit participants for blood sample eligibility screening	registry	29% (87/300) contacted provided blood samples	The use of an online registry to recruit participants to provide blood samples is feasible
Williams et al., 2023	Recruit ADRD dyads to an observational study	CE, WoM, paper mail, social media, flyer handout with home-delivered meals, brochures, PSAs, press releases, newspaper ads, newsletters, website postings	flyers with meals: 39.3%, WoM: 12.8%, paper mail: 9.4%, CE: 8.5%, newspaper ads: 8.5%, partner organization: 8.5% brochures: 6.8%, newsletter articles: 2.6%, HCP: 1.7%, website listing): 1.7% total recruitment cost/dyad: $3,148	Recruitment flyers distributed with delivered meals were the most effective strategy
Yang et al., 2024	Recruit dyads to an ADRD clinical trial	CE (community partner referrals), HCP, social media, EHRs, ads, culturally adapted material	EHRs: 82.85%, HCP: 12.59%, social media, ads, postings: 4.2%, CE: 0.03% conversion rate: 36.69% yield rate: 50.91%	Across 4 study sites, recruitment via EHRs was substantially the most effective strategy
Yu, 2013	Recruit into an ADRD study	CE, HCP, paper mail, newsletters, flyers, website, newspaper ads	HCP: 35.7%, CE: 25%, newsletters: 17.5%, flyers: 10.7%, paper mail: 7.1%, newspaper ads: 3.6% recruitment rate: 1.87	HCP referrals, followed by CE were the most effective strategies
Category 2: Studies evaluating effectiveness of recruitment of underrepresented populations to ADRD clinical research				
Briceño et al., 2020	Recruitment of Mexican American and non-Hispanic White patient/ caregiver dyads to community-based study	door-to-door case ascertainment	Mexican Americans: 60%, Non-Hispanic Whites: 35%	Door-to-door case ascertainment resulted in robust study participation
Browne et al., 2024	Recruitment of Black individuals to a population-level study	CE, HCP, recruitment company, stakeholder engagement	recruitment company: 29.7%, CE: 27.0%, stakeholder engagement: 25.7%, HCP: 16.2%	Recruitment of participants via a recruitment company, followed by CE were the most effective strategies
Byfield et al., 2023	Recruitment of African American multiplex families to genomics study	CE, WoM, print ads/flyers, radio/television interviews, community referrals	CE: 73.5%, WoM and community referrals: 13.67%, print ads/flyers and radio/television interviews: 12.7%	Culturally tailored CE via family communication was substantially most effective strategy
Chao et al., 2011	Recruitment of Chinese American individuals to ADRC	CE (health fairs), WoM, HCP, outreach clinics, print flyers/ newspapers	HCP: 36%, combined (WoM, website, flyers, newspapers, community lectures): 55.2% CE: 8.8%	HCP referrals was an effective strategy
Galvin et al., 2012	Recruitment of individuals living in rural communities to ADRC	HCP (after provider education program)	prior to program initiation: 8%, after program initiation (2000-2003): 12.8%, after program initiation (2004-2009): 13.2% increase in 30-40 participants per cycle	HCP educational programs were an effective strategy
Glover et al., 2023	Increase recruitment of diverse individuals to ADRC	CE (networking with community leaders)	Black participants: 37%, Latino participants: 10%	Engagement with community leaders was an effective strategy
Graham et al., 2018	Increase recruitment of Black individuals to clinical trials	CE (health fair, church, wellness center), WoM, HCP, paper mail, print newspapers/ ads	CE: 58.63%, print newspapers, ads: 25.68%, WoM: 6.58%, paper mail: 4.62%, HCP: 3.42%	CE via health fairs, followed by culturally tailored ads were the most effective strategies
Hinton et al., 2010	Increase recruitment of ethnic minority populations to community-based cohort	CE (screening at community events and locales)	CE: 23.58% increased recruitment conversion rate: 74.4%	CE was an effective strategy
Jacobsen et al., 2024	Increase recruitment of Black individuals to community-based cohort	CE, WoM, paper mail, flyers	CE: 43.72%, WoM: 26.12%, paper mail: 19.59%, flyer: 6.4%, registry: 3.01%, unknown: 0.5%	Combining CE and traditional ads was a successful strategy
CTAD 2023 (Study 1)	Increase recruitment of diverse participants to clinical trial	WoM, HCP, sponsored campaigns, site database	non-White participants: Site database: 54%, site campaigns: 22%, WoM (external referral): 11%, HCP: 8% White participants: site database: 29%, site campaigns: 18%, WoM (external referral): 8%, HCP: 3%	Site database was the most effective strategy
CTAD 2023 (Study 2)	Increase recruitment of underrepresented groups to a clinical trial	CE, HCP, ads	total participants: HCP: 62.16%, ads: 32.16%, CE: 5.68% underrepresented groups: CE: 86%, ads: 20%, HCP: 23%	CE was most effective for recruiting underrepresented individuals
Lee et al., 2023	Increase recruitment of Korean American individuals to clinical study	CE, WoM (at churches, senior apartments), HCP (Korean-speaking neurologist), ethnic media, social networks, ad campaign, language and race matched recruiters/ researchers	WoM: 75%, HCP: 25%	WoM was substantially most effective strategy
Li et al., 2016	Increase recruitment of Chinese American individuals to ADRC	CE, print newspaper	CE: 65.1%, newspaper: 34.9% CE cost per participant: $600 Newspaper cost: $0	CE was substantially the most effective and most costly strategy
Miller et al., 2024	Recruit underrepresented participants to ADNI4	culturally informed digital advertisements	White participants: 63% underrepresented participants (ethnocultural and educational): 42% yield rate: 21.10%	Digital advertisements was an effective strategy
Peavy et al., 2024	Increase recruitment of Vietnamese Americans into genomic study	CE, WoM (including community advisory board), paper recruitment materials	WoM: 44%, other strategies (NR)	WoM was most effective strategy
Reuland et al., 2022	Increase recruitment of Black dyads to clinical trial	CE, paper mail, email, radio and placemat ads, newsletters, articles, website, HCP	all participants: HCP: 62% White participants: HCP: 34%, paper mail (state health department): 20% Black participants: HCP: 16%, paper mail (state health department): 46%, other strategies (NR)	State health department mailing was the most effective strategy
Richards et al., 2025	Increase recruitment of Black individuals to ADRC	CE, WoM, HCP referral, social media, website, affiliated studies	Black participants: CE: 45.31%, WoM: 23.42% affiliated studies: 18.75%, social media/website: 7.8%, HCP: 4.68% non-Hispanic White participants: social media/website: 37.3%, HCP: 33.3%, WoM: 20%, affiliated studies: 6.6%, CE: 2.6% yield rate: 35.8% conversion rate: 34.9%	CE was the most effective strategy
Salazer et al., 2024	Recruit Filipino, Hispanic/Latino, and Korean individuals to AHEAD study	CE	Korean participants: 60%, Filipino participants: 32%, Hispanic/Latino participants: 8% yield rate: 10.86% conversion rate: 35.21%	CE strategies, including engagements with local organizations, was an effective strategy
Samus et al., 2015	Increase recruitment of diverse participants to a clinical trial	paper mail, registry, flyers/brochures/bookmarks, print newspaper, referrals (community liaison orgs), community outreach (radio, newspaper, community events)	all participants: paper mail: 39.6%, referrals: 29.36%, community outreach: 20.78% flyer/brochures/bookmarks: 5.6%, registry: 4.5% traditionally underrepresented participants: referrals: 44.8%, paper mail: 27.59%, community outreach: 16.1%, flyer/brochures/bookmarks: 6.89%, registry: 4.59%	Combining CE and paper mail was an effective strategy
Shaw et al., 2022	Recruitment of African American individuals to observational and prevention studies	CE (community education)	84% increased enrollment into an observational study, 52% increased enrollment into a prevention trial	Community education was an effective recruitment strategy
Walker et al., 2024	Increase recruitment of Black individuals to preclinical trials	CE, WoM (snowball sampling), paper mail, registry, social media, traditional media, speaking engagements, flyers, website	snowball sampling: 40.75%, registry: 31.1%, newspaper: 12.18%, CE: 15.96%, other strategies (NR) yield rate: 51.03% conversion rate: 74.14%	Snowball sampling was the most effective strategy
Category 3: Studies evaluating effectiveness of recruiting participants to ADRD registries				
Ajrouch et al., 2020	Recruit Middle Eastern/Arab American and Latino individuals into ADRD registries	CE, social media, paid media, newsletters	total enrollment: 217 (100 Middle Eastern/Arab American individuals enrolled, 117 Latino individuals enrolled) Recruitment goal of 120 participants exceeded	A multipronged approach, including CE was an effective strategy
Ashford et al., 2023	Recruit 5,000 Latino adults to two Brain Health Registries over 2.25 years	social media, email, search engine ads	71% of enrollment goal achieved in 12.5 months cost/enrollment: $34.25 cost/enrolled Latino individuals: $44.48 cost/enrolled Latino individuals age 55+ years old: $69.90	Culturally tailored digital advertising was an effective strategy
Gombosev et al., 2021	Recruit participants to the C2C recruitment registry	paper mail	237 participants recruitment (.27% of those contacted) cost per participant: $73 total recruitment cost: $20,000	Paper mail recruited less participants than expected into a registry and was costly
Green-Harris et al., 2019	Increase African American representation in an Alzheimer’s registry	CE	400% increase in African American representation in WARP between 2008 and 2012	CE was an effective strategy
Grill et al., 2018	Recruit participants to a local registry	CE, HCP, WoM, other referrals (partner organizations and TrialMatch), email, internet, social media, earned media	local earned media: 37%, CE: 22%, WoM: 20%, email: 8%, other referrals: 5%, internet: 5%, unknown: 5%, social media: 3%, HCP: 1% yield rate: 19.37% conversion rate: 24%	Local earned media, followed by CE, was the most effective strategy
Grill et al., 2022	Analyze recruitment sources of an online registry	CE, HCP, earned media, internet, social media, WoM, email, paper mail, not specified	email: 54.8%, CE: 9.57%, paper mail: 7.8%, not specified: 7.3%, WoM: 6.01%, internet: 4.46%, HCP: 4.42%, earned media: 3.36%, social media: 1.62%	Email was substantially the most effective strategy
Padula et al., 2024	Increase recruitment of U.S. veterans, including recruiting into registries and ADRC studies	CE, HCP, paper mail, educational videos, targeted recruitment materials	Cleveland ADRC: 5 enrolled Mount Sinai Hospital: 124 enrolled registry; 35 enrolled Stanford ADRC: 23 enrolled UCSF ADRC: 21 recruited University of Wisconsin: 48 recruited into registry	Multi-pronged recruitment methods, including CE are effective strategies
Romero et al., 2014	Recruit for a registry of research-ready, healthy older adults	CE, WoM, flyer/poster, radio/print media, website, medical center/health agencies	CE: 55.77%, radio/print media: 9.6%, website: 7.6%, medical center/health agencies: 13.3%, flyer/poster: 6.4 %, WoM: 4.5%, other sources: 2.7% yield rate: 53.83% conversion rate: 80.39%	CE was substantially the most effective strategy
Ta Park et al., 2023	Design a registry to increase Asian Americans and Pacific Islanders participation in ADRD research	CE, HCP, social media, email, newspapers, TV, university	CE: 72.4%, WoM: 10%, internet: 8%, e-mail/listserv: 4%, university: 3.4%, newspaper/TV: 1.3%, HCP: 0.2%	CE was substantially the most effective strategy
Vidoni et al., 2024	Diversify participants in an ADRC research registry	CE, WoM, HCP, social worker referral	WoM: 37.3%, CE: 35.7%, HCP: 24.7%, social worker: 0.8%, other (unspecified): 30.8%	WoM and CE were the two most effective registry strategies

Note: C2C *= consent to contact; CE = community engagement; CHC = community health clinic; EHR = electronic health records; HCP = healthcare provider referral; NR = not reported; PSA = public service announcement; WoM = word of mouth*

Four studies reporting general recruitment strategies that utilized CE methods—including ADRD-related presentations, memory screenings, health fairs, and the use of community health centers as recruitment sites—found that CE was the most effective strategy. In these studies, CE recruited 57-100% more participants than other recruitment strategies [39], [40], [81], [83].

HCP and WoM referrals were deemed effective recruitment strategies. One study reported that the combination of WoM and HCP referrals was the second most effective strategy after CE (28.1% of participants). Similarly, another study found that HCP referrals were the most effective strategy among those tested (35.7% of participants), followed by a CE informational presentation (25% of participants) [42,80]. Two of the 5 articles that employed HCP or WoM referrals found that WoM recruiting was the third most effective strategy, compared with broadcast media, print media, and identifying participants from electronic health records (EHRs)/registries/memory clinic rosters (34.2% and 6.1% of participants, respectively) [45,50].

The effectiveness of direct mailing as a recruitment strategy showed mixed results. Two studies found direct mailing to be the most effective approach, yielding over half of all participants enrolled [43,45], whereas 5 studies reported that it was less effective than other multi-pronged strategies [39], [42], [52], [80], [83]. For example, one study found that geo- and age-targeted postcards and brochures generated 52% of enrollment, and another study achieved 39.3% of enrollment through flyers distributed with home-delivered meals, out-performing other strategies [40,45]. Several studies (n=5) leveraged other print media (e.g., flyers, brochures, newspaper advertisement, magazines) to recruit participants, though the effectiveness of these strategies varied across studies [42,43,50,52,80].

Six studies used social media as a recruitment strategy, with mixed results. One study assessed its effectiveness as a standalone strategy [78], whereas the others used it within a multi-pronged recruitment approach [26,42,47,53,54]. One study examined the number of followers to their ADRD topic-specific social media accounts over time and subsequent enrollment in Alzheimer’s Disease Research Center (ADRC) studies [78]. Of the 76 individuals who expressed interest—via a webform or phone screen—to participate in ADRC studies, 61% (n=46) were referred to an ongoing research study, highlighting the potential of social media to supplement other recruitment strategies [78]. Another study reported that a social media campaign was the most effective recruitment strategy for their study, accounting for 86% of enrolled participants and outperforming all original strategies combined (i.e., referrals, mailings, advertisements, community outreach) [54]. In contrast, two other studies reported minimal impact: one found that the combination of social media, the study website, and research registries enrolled only 3 participants (3.1% of enrollment), and another reported that social media accounted for 2.8% of participants [26,42].

Other less commonly used multi-pronged approaches included help lines [72]; patient information databases (e.g., EHRs, memory clinic patient rosters) [45,46]; websites [26,47,80]; TV and radio advertisements [45]; and list serve emails [47]. Notably, one study reported that websites across seven recruitment sites accounted for 50.9% of participant enrollment [26]. Two studies found that the Consent-to-Contact (C2C) Registry yielded enrollment rates of 26% and 28.9% across multiple studies [64,71]. In another study, a registry was used to identify and recruit participants for blood sample collection, screening, and potential referrals to preclinical trials. Among those contacted through the registry, 29% provided blood samples [31].

The effectiveness of recruitment strategies varied across studies, and most incorporated a myriad of approaches in their recruitment process. The widespread use of multi-pronged recruitment approaches reflects the diversity of ADRD populations and underscores the need for a comprehensive portfolio of recruitment methods that can be tailored to specific enrollment goals.

#### 3.2.2 Increasing recruitment for underrepresented groups

The 20 studies in this category (40%) reported success in increasing recruitment of underrepresented populations, although the effectiveness of specific strategies varied across groups. CE generally outperformed other strategies, enrolling more participants than WoM, HCP referrals, phone calls, advertisements (e.g., newspapers, radio), email, and recruitment from affiliated studies (Table 1) [40,57,73,75,77]. Two studies effectively implemented culturally tailored strategies (e.g., culturally sensitive language, religious beliefs, dietary preferences) [21,73], which helped build trust between community members and study teams, and enhanced recruitment [21,55]. The effectiveness of CE varied across racial/ethnic groups, recruiting more Black/African American participants (45.31% and 37%) compared with White non-Hispanic (36%) and Latino (10%) participants in the respective studies [59,77]; and more Korean participants (60%) compared with Filipino and Hispanic/Latino participants (32% and 8%, respectively) [59,77]. Furthermore, two studies found that HCP referrals were less effective for recruiting Black/African American participants, including patient-caregiver dyads (16% and 4.68%), than for White participants (34% and 33.3%), although one study reported HCP referral success in recruiting Asian American participants (36% of participants) [51,58,77].

Several notable exceptions to CE effectiveness emerged. One study found that snowball sampling—a method in which current participants aid in recruitment by referring others from their social networks to a study—and research registry recruitment outperformed CE strategies in recruiting Black participants to an ADRD observational study (40.75% versus 15.96% of participants, respectively) 84. Another study partnered with community organizations to send letters to clients, yielding 39.6% of all enrollees and 27.59% of underrepresented enrollees—outperforming community outreach (CE events and advertisements) in both groups (20.78% and 16.1%, respectively) [44]. WoM was the most effective strategy for recruiting Korean and Vietnamese American populations, compared to CE and HCP referrals [61,85]. Notably, one study enhanced a WoM strategy by leveraging a community-based information system to map the geographic distribution and sociodemographic characteristics of Korean American individuals. This approach enabled the identification of optimal geographic locations for recruitment, successfully enrolling 75% of participants [61].

Other recruitment strategies included the use of an online database to recruit “ethnically diverse” participants, recruiting more non-White participants (54% of participants) than White participants (18% of participants) [56]; educating HCPs in rural areas to increase dementia awareness, which increased HCP referrals from 8% to 13.2% [79]; and partnering with third-party companies to recruit Black/African American participants, which yielded two additional participants beyond those recruited through CE strategies [73]. Among less common approaches, one study reported that door-to-door participant ascertainment in a local community and in nursing homes resulted in robust participation of dyads among Mexican American populations [49]. Another study reported that culturally informed digital advertisements successfully recruited underrepresented participants—defined by ethnocultural background and educational attainment—resulting in a sample consisting of 42% underrepresented individuals [48].

Overall, many CE efforts evaluated in this review were considered successful in increasing recruitment of underrepresented groups. However, recruitment strategies were not equally effective across all groups, underscoring the importance of culturally sensitive approaches when aiming to increase participation among underrepresented populations. Variation in how studies reported recruitment outcomes, the frequent use of multi-pronged approaches, and the broad definition of what constitutes community engagement make it difficult to draw firm conclusions about CE’s comparative effectiveness and highlight the need for further investigation.

#### 3.2.3 Recruiting for registries

Participant registries are increasingly used in ADRD clinical research [68]. These registries enable researchers to rapidly contact large numbers of potentially eligible participants and to target outreach efforts based on demographics [64]. As a result, recent efforts have focused on expanding and diversifying registry enrollment. Ten studies (20%) reported recruitment efforts specifically aimed at increasing registry enrollment (Table 1) [29,62,63,[65], [66], [67], [68], [69], [70],^76^]. Although the primary goal of established registries is to expand the ADRD participant pool, several studies in this review emphasized efforts within registries to improve the representation of racial/ethnic groups, including Middle Eastern/Arab American, Asian American and Pacific Islander, African American, Latino, and veteran populations [62,63,65,67,76].

Most of the studies (n=8) employed multi-pronged approaches to recruit participants into ADRD registries. Three of these identified CE as the most effective component of their recruitment strategy [[65], [66], [67]]. Notably, one study using a community-based participatory research approach that included CE in a multi-pronged recruitment strategy exceeded its original registry enrollment goals [62]. Moreover, an asset-based community development model increased African American participation in the Wisconsin Registry for Alzheimer’s Project by 400% [65]. In contrast, one study reported that direct mailings followed by calls were more effective than CE for recruiting veterans, and another reported that local earned media outperformed CE in recruiting participants (37% and 22%, respectively) [69].

Other strategies for recruiting participants into registries produced mixed results, including referrals (HCP, social worker, WoM) [29,[66], [67], [68], [69]], email [29,63,67,69], social media campaigns [62,63], paper mail [29,70], and promotion through flyers and event promotion tables [68]. Notably, one study found that email was the most effective strategy of those tested, accounting for more than half of participants, while others reported that email generated only 8% and 4% of participants, respectively [29,67,69]. The effectiveness of paper mail also varied, recruiting 0.27% of those contacted in one study (fewer than researchers anticipated), compared with 7.8% participants in another [29,70]. One study relied exclusively on social media and other digital tools (i.e., websites, emails, digital media advertisements) for registry recruitment and was successful in enrolling Latino participants, achieving 71% of its enrollment goal in 12.5 months [63].

The evidence base on recruiting participants into ADRD-focused registries is still developing. However, registry recruitment strategies generally mirror those used for direct recruitment into ADRD clinical studies—often involving multi-pronged approaches that include CE, with effectiveness varying across strategies.

### 3.3 Appraisal of study quality

Included studies were assessed for quality within their methodological domain. Only the three published poster presentations included in the review lacked sufficient information for appraisal [55,56,71]. For this review, the MMAT was used to evaluate the quality of included studies rather than to exclude studies, which is its common use in systematic reviews. MMAT scores for included studies ranged from 2 (meeting 40% of study quality criteria) to 5 (meeting 100% of study quality criteria).

Overall, the 47 assessed studies had a mean MMAT score of 4 (80%), indicating that there is “room for improvement” in study quality (Table 2). Studies often lacked sufficient information to determine if they met a criterion, receiving a “cannot tell” or 0 for that criterion, which lowered their individual score and the subsequent average score of all included studies. For example, many quantitative descriptive studies did not provide sufficient information to assess representative samples (53.3%) and non-response bias (51.1%). Studies examining recruitment methods may be particularly susceptible to non-response bias, as participants are generally not randomized to a specific recruitment strategy.

**Table 2:** Mixed methods appraisal tool (MMAT) - study quality appraisal.

Author, Year	Screening		Non-randomized studies					Quantitative descriptive studies					Score
	S1	S2	3.1	3.2	3.3	3.4	3.5	4.1	4.2	4.3	4.4	4.5	
Ajrouch 2020	Yes	Yes						Yes	Yes	Yes	Can't tell	Yes	80% (4)
Ashford 2023	Yes	Yes	No	Yes	Yes	Can't tell	Yes						60% (3)
Austrom 2010	Yes	Yes						Yes	Can't tell	Yes	Can't tell	Yes	60% (3)
Birnbaum 2025	Yes	Yes						Yes	Yes	Yes	Yes	Yes	100% (5)
Briceno 2020	Yes	Yes						Yes	Yes	Yes	Yes	Yes	100% (5)
Browne 2024	Yes	Yes						Yes	Yes	Can't tell	Can't tell	Can't tell	40% (2)
Byfield 2023	Yes	Yes						Yes	Yes	Can't tell	Yes	Yes	80% (4)
Carr 2010	Yes	Yes	Yes	Yes	Yes	Can't tell	Can't tell						60% (3)
Chao 2011	Yes	Yes						Yes	Yes	Yes	Yes	No	80% (4)
Cowie 2018	Yes	Yes						Yes	No	Yes	No	Yes	60% (3)
Galvin 2012	Yes	Yes						Yes	Can't tell	Yes	Can't tell	Yes	60% (3)
Glover 2024	Yes	Yes						Yes	Can't tell	Yes	Yes	Yes	80% (4)
Gombosev 2021	Yes	Yes						Yes	No	Yes	No	Yes	60% (3)
Graham 2018	Yes	Yes						Yes	Yes	Yes	Can’t tell	Yes	80% (4)
Green-Harris 2019	Yes	Yes	No	Can't tell	Yes	Can't tell	Yes						40% (2)
Greimel 2022	Yes	Yes						Yes	Can't tell	Yes	Yes	Yes	80% (4)
Grill 2022	Yes	Yes						Yes	Yes	Yes	Yes	Yes	100% (5)
Grill 2018	Yes	Yes						Yes	Can’t tell	Yes	Yes	Yes	80% (4)
Hinton 2010	Yes	Yes	Yes	Yes	Yes	Yes	Yes						100% (5)
Jacobsen 2024	Yes	Yes						Yes	Yes	Yes	Yes	Yes	100% (5)
Keohane 2023	Yes	Yes						Yes	No	Yes	Can't tell	Yes	60% (3)
Kirn 2023	Yes	Yes						Yes	Can't tell	Yes	Yes	Yes	80% (4)
Lee 2023	Yes	Yes						Yes	Yes	Can't tell	Can't tell	Yes	60% (3)
Li 2016	Yes	Yes						Yes	Yes	Yes	Yes	Yes	100% (5)
Melikyan 2019	Yes	Yes						Yes	No	Yes	Yes	Yes	80% (4)
Miller 2024	Yes	Yes						Yes	No	Yes	No	Yes	60% (3)
Morrison 2015	Yes	Yes						Yes	No	No	No	Yes	40% (2)
Naylor 2024	Yes	Yes						Yes	Can't tell	Yes	Can't tell	Yes	60% (3)
Padula 2024	Yes	Yes						Yes	Yes	Yes	Yes	Yes	100% (5)
Peavy 2020	Yes	Yes						Yes	Can't tell	Yes	Yes	Yes	80% (4)
Peavy 2024	Yes	Yes						Yes	Yes	Yes	Yes	Yes	100% (5)
Reuland 2022	Yes	Yes						Yes	Yes	Yes	Yes	Yes	100% (5)
Richards 2025	Yes	Yes						Yes	No	Yes	Can't tell	Yes	60% (3)
Romero 2014	Yes	Yes						Yes	Yes	Yes	Yes	Yes	100% (5)
Samus 2015	Yes	Yes						Yes	Yes	Yes	Can't tell	Yes	80% (4)
Shadyab 2021	Yes	Yes						Yes	Can’t tell	Yes	Yes	Yes	80% (4)
Shaw 2022	Yes	Yes	Yes	Yes	No	Can’t tell	Yes						60% (3)
Szabo-Reed	Yes	Yes						Yes	Can't tell	Yes	Can't tell	Yes	60% (3)
Ta Park 2023	Yes	Yes						Yes	Yes	Yes	Yes	Yes	100% (5)
Teano 2024	Yes	Yes						Yes	Can't tell	Yes	No	Yes	60% (3)
Vidoni 2024	Yes	Yes						Yes	Yes	Yes	Yes	Can't tell	80% (4)
Walker 2024	Yes	Yes						Yes	Yes	Yes	Yes	Yes	100% (5)
Walter 2024	Yes	Yes						Yes	Can't tell	Yes	Can't tell	Yes	60% (3)
Walter 2020	Yes	Yes						Yes	Can't tell	Yes	No	Yes	60% (3)
Williams 2023	Yes	Yes						Yes	Can't tell	Yes	Can't tell	Can't tell	40% (2)
Yang 2024	Yes	Yes						Yes	Can't tell	Yes	Can't tell	Can't tell	40% (2)
Yu 2013	Yes	Yes						Yes	Can't tell	Yes	Can’t tell	Yes	60% (3)

Beyond MMAT scores, inconsistencies in reported outcomes further limited the ability to compare recruitment strategies for effectiveness. For example, 94% of studies reported only the total number of participants enrolled, while few included outcome measures such as conversion rate (14%), yield rate (18%), or recruitment rate (4%). Relatively few studies reported on recruitment time or financial costs (22%). In many cases, these gaps may reflect the retrospective nature of the analyses conducted on existing data, rather than studies designed to evaluate recruitment strategy efficacy. The lack of planning for recruitment strategy analysis in the original study designs likely contributed to limited reporting beyond total enrollment and, in some cases, lower MMAT scores. Overall, these quality appraisal findings indicate that many studies lacked a systematic approach to reporting data. Given the high number of “cannot tell” decisions across domains, it remains unclear whether these reflect omissions in reporting or inherent limitations in study design.

### 3.4 Limitations

One of the main challenges encountered in this review was identifying articles that documented effective and ineffective approaches to enhancing recruitment for ADRD clinical trials. Recruitment information is often embedded within broader clinical trial publications making it difficult to identify studies focused specifically on recruitment strategies. For example, traditional search strategies using search terms such as “clinical research/trial” and “recruitment” generated an unmanageable number of irrelevant results focused on clinical trial results rather than reporting on recruitment outcomes specifically. Conversely, with a more refined search approach there was risk of omitting important studies. To address this, targeted searches were conducted in PubMed, Google Scholar, and predetermined websites. These searchers were supplemented by citation searching of literature reviews, identified papers, and publications of leading authors in the field.

Another limitation was the variability in how recruitment strategy effectiveness was reported. This heterogeneity complicates the assessment of recruitment effectiveness, particularly when strategies are employed across different participant groups with limited outcome data. Where available, attempts were made to compare yield and conversion rates, calculated directly from data provided in study tables and text; however, so few studies provided this information that comparisons across strategies were limited.

The MMAT framework was used to assess study quality. Although the MMAT developers provide a scoring algorithm that has been widely adopted in the literature, they recommend generating summaries of study quality that reflect the raw MMAT criteria ratings rather than relying solely on aggregated scores, which assign equal weight to all MMAT criteria [86]. Accordingly, Table 2 reports scores for each MMAT domain as well as the total and percentage scores.

Lastly, due to gaps in the literature, this review was unable to characterize potentially important differences in effective recruitment strategies for clinical trials conducted at both academic and commercial sites. Despite the considerable number of ongoing clinical trials at both types of sites [87], only four included studies reported on recruitment methods in pharmacological trials. Only 15 clinical trials conducted at academic sites reported on direct (non-registry) recruitment strategies. These findings suggest that a substantial amount of valuable data on effective recruitment approaches in ADRD clinical trials, whether conducted at commercial or academic sites, remains unpublished.

## 4 Discussion

Participant recruitment and retention have been described as the “greatest obstacle” to developing new AD treatments [14]. Recognizing this challenge, this review aimed to synthesize the literature to discern effective and ineffective recruitment approaches. A key finding of this review is that the literature on recruitment strategies for ADRD clinical trials is both limited and highly heterogeneous. The literature is limited in part because many studies reporting on recruitment strategy efficacy are conducted in the context of non-pharmacologic ADRD research or participant registries, rather than pharmacologic treatment trials. Yet accelerating pharmacologic treatment development requires understanding recruitment in the relevant trial setting.

At the same time, the literature encompasses a wide variety of recruitment strategies. This heterogeneity, combined with the use of multi-pronged recruitment approaches, inconsistent outcome reporting, and limited information on recruitment time and cost, constrains the ability to draw definitive conclusions about the effectiveness of specific recruitment strategies. While some of this variability may be appropriate, reflecting the need to tailor recruitment strategies to different populations and settings, much of the documented variation likely stems from the limited empirical evidence available to guide recruitment design. Nonetheless, several patterns were identified.

Many studies reported CE as either a standalone strategy or part of multi-pronged recruitment approaches. CE efforts inform potential participants about research opportunities and participatory significance, while also fostering long-term community partnerships through collaboration. CE strategies can be culturally tailored, providing information in participants’ preferred languages and ensuring cultural relevance to reduce participation barriers. Although CE strategies vary (e.g., health fairs, memory screenings, presentations at churches and wellness centers), their widespread use suggests CE is widely viewed as an effective strategy for recruiting hard-to-reach ADRD populations. Further research is needed to determine which CE approaches are most effective in different contexts and populations, and whether they are similarly effective for ADRD clinical trial recruitment across diverse communities.

Social media campaigns are also an emerging recruitment strategy, demonstrating effectiveness for both broad populations and specific underrepresented groups. Structured and well-planned campaigns tend to be more successful than general social media use, challenging assumptions that older adults in ADRD research are difficult to reach through these platforms. The adaptability of such technological solutions offers promise for overcoming recruitment obstacles across diverse research contexts and provides potentially low-cost ways for systematically capturing recruitment data.

Across all recruitment approaches, whether CE, social media, or other strategies, future efforts should measure not only recruitment effectiveness but also the associated time and financial costs. Understanding how strategies compare in terms of the cost per additional enrollee, or per additional enrollee from specific priority groups, can help clinical trials allocate recruitment resources more efficiently and make more informed decisions about which approaches to scale. The knowledge gaps identified in this review suggest two critical paradigm shifts are necessary to advance ADRD recruitment research as a mature field: [1] the routine and transparent reporting of recruitment strategies used in clinical trials, along with their associated costs, within an evidence-based framework; and [2] the widespread adoption and reporting of quantitative recruitment outcome measures.

The first paradigm shift addresses the lack of information on clinical trial recruitment sponsored by pharmaceutical companies and academia, as recruitment data from both sectors remain largely unpublished. ADRD pharmacological trials face significant recruitment challenges compared with non-pharmacological trials, largely due to numerous exclusion criteria and study procedures that can limit real-world and underserved representation [25]. These recruitment barriers are particularly concerning because ADRD pharmacological trials already experience slower enrollment and higher costs than trials in other therapeutic areas [12,22,87]. The consequences of slow enrollment are substantial: it increases trial costs, extends study duration, delays potential patient benefit, and can compromise statistical power—potentially resulting in inconclusive findings about drug efficacy due to inadequate sample sizes. Collectively, these factors hinder the speed of ADRD drug development [11,13,88,89].

The biopharmaceutical industry, which sponsors 62% of all AD clinical trials, is uniquely positioned to address this information gap [87]. Currently, industry sponsors may deprioritize the reporting of recruitment outcomes due to the costs and effort associated with publication. Emphasizing the benefits of transparent reporting is critical, as it offers mutual advantages for both industry stakeholders and the broader research community. Widespread reporting of recruitment outcomes would allow future trials to access effective strategies, helping to overcome slow enrollment barriers. These reported outcomes could then inform recruitment planning and study design, ultimately reducing costs associated with delayed or halted trials and strengthening the overall drug development pipeline [10,11]. Academic-sponsored clinical trials were also underrepresented in this review. Increasing data sharing in this sector could involve identifying and encouraging incentives for reporting recruitment outcomes. For example, because academic clinical trials often rely on grant funding from agencies such as the National Institutes of Health (NIH) [21,31,44,54,83], these agencies could require and provide funding to support the reporting of standardized recruiting metrics as part of their grant provisions.

The clinical trial sector could champion recruitment research by embedding preplanned data collection and analysis of recruitment into their trial protocols, with the intent of publicly sharing the results, as recommended in the Clinical Trials Transformation Initiative (CTTI) Framework for Strategic Recruitment Planning [90]. Routinely reporting recruitment data in publications—either as supplementary materials or in compendiums accompanying trial results—would ensure that the data are widely accessible. Further, well-informed recruitment and study design should prioritize representative samples to ensure generalizable study results [91]. By setting a standard for recruitment reporting, both industry sponsors and academia can catalyze systematic, nationwide improvements across all pharmacological and biomarker studies.

The second paradigm shift involves implementing measures to address the lack of standardized reporting of recruitment outcomes. As noted, synthesizing data and drawing conclusions about the effectiveness of recruitment strategies was challenging in this review due to inconsistent outcome reporting, a limitation also noted in previous ADRD recruitment literature reviews [18,34,35]. Advancing the science of ADRD recruitment requires consistent reporting of recruitment efficacy across contexts, including successes and failures, as well as the associated costs of recruitment strategies.

Formal guidelines for recruitment outcome reporting in ADRD research could help achieve these goals. A practical path toward standardization would involve convening a multi-stakeholder working group to establish shared definitions and a common vocabulary for recruitment metrics. Using a structured consensus process, such a group could identify a minimum core set of recruitment outcomes recommended for reporting, supported by standardized reporting templates to facilitate implementation across studies. However, guidelines alone are unlikely to be sufficient. Aligned incentives, such as funder requirements and journal expectations, will likely be needed to ensure widespread adoption. Furthermore, given tight research budgets, dedicated funding to support standardized recruitment data collection and reporting within real-world trials is essential to build an evidence base on recruitment effectiveness. Ultimately, this work could inform the design of more efficient ADRD clinical trial recruitment strategies.

In response to the limited evidence base on recruitment science and growing recognition of its importance, recent work has sought to build more systematic knowledge. For example, a recent randomized controlled trial by Jacobson et al. (2025) exemplifies a rigorous evaluation of recruitment methods that can be replicated by others [92]. To recruit participants into a memory concerns registry, potential participants were randomly assigned to receive one of three messages: a simple invitation (control), an invitation offering a guaranteed $25 incentive, or an invitation offering a 1-in-100 chance at $2,500. Both recruitment rates and costs were measured for each strategy. While the guaranteed $25 incentive produced the highest recruitment rate, the message-only approach was the most cost-efficient. Notably, financial incentives increased recruitment overall but did not enhance participant diversity. Another recent study by Ritchie et al. (2025) reported clear comparisons of recruitment strategies between clinical trials at academic sites and non-academic sites, finding that academic sites recruited more participants with earned media and referrals from organizations (e.g., Alzheimer’s Association, NIH) and fewer participants through advertisements and referrals from site staff and databases [93].

Others have made important strides in systematic data capture, such as the ACTC, which established a pre-screening database and a minimal dataset used across all ACTC trials. This system captures information on participant recruitment, including calls to potential participants, referrals, and responses to recruitment campaigns, enabling analysis of recruitment strategies effectiveness across different trials [26,94]. The database also includes reasons for pre-screen ineligibility, as well as demographic information such as ethnicity and sex —critical data for evaluating recruitment strategies aimed at enrolling diverse participants [26].

The Alzheimer’s Association supports recruitment efforts through its nationwide chapter network and the TrialMatch initiative [3]. TrialMatch is a free, web-based service that connects individuals with AD, including those with early-onset AD, to clinical trials based on their diagnosis, stage of disease, and location. Such organizational support plays a vital role in bridging gaps between participants and research teams, enhancing recruitment capabilities. Beyond established programs like TrialMatch, emerging technology-driven strategies may help advance the science of recruitment [22]. Digital platforms—including online engagement tools, social media channels, telehealth services, and EHRs—have significantly expanded the reach of recruitment efforts by providing new avenues for connecting potential participants.

## 5 Conclusion

Nearly a decade after the launch of the NIA’s National Strategy on Recruitment and Retention in ADRD research, the applied science of recruitment remains an emerging field. Despite growing attention and concerted efforts, foundational evidence on effective recruitment strategies, particularly for pharmacological trials, remains limited. This evidence gap constrains the ability of stakeholders, including researchers, private industry, and government funders, to evaluate, optimize, and adopt improved strategies. Strengthening the evidence base requires a shift from ad hoc analyses and inconsistent reporting toward systematic, standardized practices. Standardized reporting will enable comparisons and replication of successful ADRD recruitment strategies, facilitate the development of generalizable findings inclusive of underrepresented populations, and accelerate progress in ADRD prevention and treatment. The existing literature provides important insights that can serve as the foundation for advancing recruitment science, and collecting standardized metrics will further strengthen the field.

## Funding

This work was supported by Gates Ventures.

## Declaration of generative AI and AI-assisted technologies in the writing process

During the preparation of this work the authors used OpenAI’s ChatGPT in order to improve the readability of the manuscript. After using this tool, the authors reviewed and edited the content as needed and take full responsibility for the content of the published article.

## Ethical statement

Not applicable.

## Data statement

Not applicable.

## CRediT authorship contribution statement

**Mireille Jacobson:** Writing – review & editing, Writing – original draft, Supervision, Methodology, Conceptualization. **Christina Deuschle:** Writing – review & editing, Writing – original draft, Visualization, Project administration, Methodology, Investigation, Conceptualization. **Desi Peneva:** Writing – review & editing, Writing – original draft, Supervision, Methodology, Funding acquisition, Conceptualization. **Alice Nuo-Yi Wang:** Writing – review & editing, Writing – original draft, Visualization, Investigation, Data curation. **Cooper Roache:** Writing – review & editing, Writing – original draft, Visualization, Investigation, Data curation. **Meghan Walsh:** Writing – review & editing, Writing – original draft, Visualization, Supervision, Methodology, Investigation, Conceptualization. **Phyllis Barkman Ferrell:** Writing – review & editing, Supervision, Methodology, Conceptualization. **Maria-Alice Manetas:** Writing – review & editing, Project administration, Conceptualization. **Rema Raman:** Writing – review & editing, Supervision. **Paul Aisen:** Writing – review & editing, Supervision. **Dana Goldman:** Writing – review & editing, Supervision, Funding acquisition.

## Declaration of competing interest

The authors declare the following financial interests/personal relationships which may be considered as potential competing interests:

Mireille Jacobson reports grants from National Institutes of Health and American Heart Association. Christina Deuschle, Alice Nuo-Yi Wang, Cooper Roache and Meghan Walsh report no conflict of interest. Desi Peneva and Maria-Alice Manetas report financial support for this work provided by Gates Ventures. Phyllis Barkman Ferrell reports consulting or advisory relationship with Adams Clinical, Davos Alzheimer's Collaborative, Global Alzheimer's Platform Foundation, SiteRx. Rema Raman reports grants from Alzheimer's Association, American Heart Association, Eisai, National Institute on Aging. Paul Aisen reports grants from Alzheimer's Association, Lilly, National Institutes of Health; consulting or advisory relationship with AbbVie, Arrowhead Pharmaceuticals, Biogen, Checkpoint Therapeutics, Genentech, Immunobrain, Merck, Roche; and research collaboration with Eisai and Cognition Therapeutics. Dana Goldman reports grants from Alexion, American Heart Association, Amgen, Biomarin Pharmaceutical, Blue Cross Blue Shield of Arizona, Blue Cross Blue Shield of Massachusetts, BrightFocus, Bristol Myers Squibb, California Hospital Association, Cedars-Sinai Health System, Charles Koch Foundation, CommonSpirit, Edwards Lifesciences, Gates Ventures, Genentech, Gilead Sciences, Incyte, Johnson & Johnson, Lilly, National Institute on Aging, National Institute of Diabetes and Digestive and Kidney Diseases, Novartis, Pfizer, RA Capital, Sarepta Therapeutics; consulting or advisory relationship with Edwards Lifesciences and GRAIL; equity in EntityRisk.

## Appendix Supplementary materials

Image, application 1
