# Supplementary material for: Evaluating evidence-based recruitment strategies for Alzheimer’s disease and related dementias clinical trial research: A literature review
Source: J Prev Alzheimers Dis. 2026 Mar 14;13(5):100532. doi: 10.1016/j.tjpad.2026.100532 (PMC12999305; doi:10.1016/j.tjpad.2026.100532)
Supplement: Supplementary file 1 [file mmc1.docx]

Evaluating Evidence-Based Recruitment Strategies for Alzheimer's Disease and Related Dementias Clinical Trial Research: A Literature Review

Supplementary Material

**Table 1.** Organization websites included in literature search

| Organization Name | Organization Type |
| --- | --- |
| AARP Global Brain Health Council | Independent collaborative |
| Alzheimer’s and Dementia Patient and Caregiver Powered Research Network | Research collaboration (by UsAgainstAlzheimer’s) |
| Alzheimer’s Association | Nonprofit |
| Alzheimer’s Clinical Trials Consortium | Multi-principal investigator consortium |
| BeingPatient | Community platform |
| Biogen | Biotechnology company |
| BrightFocus Foundation | Nonprofit |
| Care Access | Clinical research organization |
| CDC Healthy Brain Initiative | Government agency initiative |
| Charter Research | Clinical research organization |
| Clariness | Patient recruitment and retention company |
| Clinical Trials Media (CTM) | Patient recruitment and retention company |
| FNIH Accelerating Medicines Partnership Program Alzheimer’s Disease | Public-private partnership |
| Global Alzheimer’s Platform Foundation | Nonprofit |
| IQVIA | Clinical research organization |
| K2 Medical Research | Clinical research organization |
| Lilly | Pharmaceutical company |
| Lotus (Clinical Research, LLC) | Clinical research organization |
| National Alzheimer’s Coordinating Center | Government-funded center |
| National Institute on Aging (NIA) | Government agency |
| Parexel | Clinical research organization |
| RAND Corporation | Nonprofit |
| SiteRX | Healthcare information technology company |
| UsAgainstAlzheimer’s | Nonprofit |
| Women’s Alzheimer’s Movement | Nonprofit |
| Women’s Health Access Matters | Nonprofit |

Note: All websites were searched for research meeting inclusion criteria.

**Table 2.** Included studies reporting recruitment, yield, and conversion rate with corresponding MMAT scores

| Reported Recruitment Outcome Measures | | | | | |
| --- | --- | --- | --- | --- | --- |
| Study | **Recruitment Strategies** | **Recruitment Rate** | **Yield Rate** | **Conversion Rate** | **MMAT Score** |
| Greimel et al., 2022 | CE; WoM; HCP; paper mail; social media; registry; other (press releases, flyer/brochure, newspaper/magazine, website) | 2.14 | 76.20 | 31.90 | 80% (4) |
| Hinton et al., 2010 | CE | NA | NA | 74.50 | 100% (5) |
| Richards et al., 2025 | CE; WoM; HCP; other (community book drive) | NA | 35.80 | 34.90 | 60% (3) |
| Romero et al., 2014 | CE; WoM; other (flyer/poster, radio/newspaper/newsletter articles, website, medical center) | NA | 53.83 | 80.39 | 100% (5) |
| Shadyab et al., 2021 | CE; paper mail; registries; EHRs; other (memory clinic rosters, news broadcasts, PSAs, local ads) | NA | NA | 29.80 | 80% (4) |
| Szabo-Reed et al., 2023 | Email; other (interviews/ editorials, print ads, broadcast advertising, print marketing, website, display advertising); registry; HCP; WoM (another participant, personal contact); paper mail; CE (community presentations); social media | NA | NA | 63.73 | 60% (3) |
| Walker et al., 2024 | CE; WoM; paper mail; registry; social media; other (traditional media, speaking engagements, flyers, website) | NA | 51.03 | 74.14 | 100% (5) |
| Walter et al., 2024 | Registry | NA | 29.00 | NA | 60% (3) |
| Yang et al., 2024 | CE; HCP; social media; EHRs; Other (ads, culturally adapted material) | NA | 50.91 | 36.68 | 40% (3) |
| Yu, 2013 | CE; HCP; paper mail; other (Alzheimer's Association newsletters, flyers, website, newspaper ads) | 1.87 | 32.90 | 57.14 | 60% (3) |

Note: CE = community engagement; EHRs = electronic health records; HCP = healthcare provider referral; PSAs = public service announcements; WoM = word of mouth. Table summarizes all studies that reported recruitment, yield, or conversion rates or provided data enabling calculation of these rates, along with their MMAT scores.
